# A Novel Fault Detection Method for Rolling Bearings Based on Non-Stationary Vibration Signature Analysis

**DOI:** 10.3390/s19183994

**Published:** 2019-09-16

**Authors:** Dong Zhen, Junchao Guo, Yuandong Xu, Hao Zhang, Fengshou Gu

**Affiliations:** 1 Tianjin Key Laboratory of Power Transmission and Safety Technology for New Energy Vehicles, School of Mechanical Engineering, Hebei University of Technology, Tianjin 300401, China; jc_guo12@163.com; 2 Centre for Efficiency and Performance Engineering, University of Huddersfield, Huddersfield HD1 3DH, UK; yuandong.xu@hud.ac.uk (Y.X.); f.gu@hud.ac.uk (F.G.)

**Keywords:** weighted average ensemble empirical mode decomposition, modulation signal bispectrum, Teager energy kurtosis, fault detection, rolling element bearing

## Abstract

To realize the accurate fault detection of rolling element bearings, a novel fault detection method based on non-stationary vibration signal analysis using weighted average ensemble empirical mode decomposition (WAEEMD) and modulation signal bispectrum (MSB) is proposed in this paper. Bispectrum is a third-order statistic, which can not only effectively suppress Gaussian noise, but also help identify phase coupling. However, it cannot effectively decompose the modulation components which are inherent in vibration signals. To alleviate this issue, MSB based on the modulation characteristics of the signals is developed for demodulation and noise reduction. Still, the direct application of MSB has some interfering frequency components when extracting fault features from non-stationary signals. Ensemble empirical mode decomposition (EEMD) is an advanced nonlinear and non-stationary signal processing approach that can decompose the signal into a list of stationary intrinsic mode functions (IMFs). The proposed method takes advantage of WAEEMD and MSB for bearing fault diagnosis based on vibration signature analysis. Firstly, the vibration signal is decomposed into IMFs with a different frequency band using EEMD. Then, the IMFs are reconstructed into a new signal by the weighted average method, called WAEEMD, based on Teager energy kurtosis (TEK). Finally, MSB is applied to decompose the modulated components in the reconstructed signal and extract the fault characteristic frequencies for fault detection. Furthermore, the efficiency and performance of the proposed WAEEMD-MSB approach is demonstrated on the fault diagnosis for a motor bearing outer race fault and a gearbox bearing inner race fault. The experimental results verify that the WAEEMD-MSB has superior performance over conventional MSB and EEMD-MSB in extracting fault features and has precise and effective advantages for rolling element bearing fault detection.

## 1. Introduction

Rolling element bearings have been widely used in modern industries, but failure may lead to fatal breakdowns and costly downtime [1]. Whether its running status is normal or not directly affects the efficiency and performance of the whole machine system [2]. Without early warning, bearing failures would lead to economic losses and serious security problems. Therefore, rolling element bearing failures are essential to ensure the normal operation of the mechanical system at their incipient stage and have received much attention in recent years [3,4,5].

Currently, many advanced signal processing methods have been developed for bearing fault detection based on vibration signature analysis, such as Wigner–Viller distribution (WVD), empirical wavelet transform (EWT), wavelet packet transform (WPT), local mean decomposition (LMD), variational mode decomposition (VMD), multivariate empirical mode decomposition (MEMD), and masking empirical mode decomposition (EMD), etc. Although these methods are effective in detecting the failures of rolling element bearings, they still have some limitations. For instance, the WVD has a higher time–frequency resolution, but it is restricted by cross term interference [6,7]. The EWT is an excellent signal decomposition method, but it suffers from the binary band allocation [8,9]. The WPT has good de-noising performance, but the basic functions need to be provided in advance [10,11]. The LMD is an adaptive analysis method for non-stationary signals, but it is affected by mode mixing [12,13]. The VMD is an adaptive and quasi-orthogonal signal decomposition approaches, but its penalty parameter and decomposition number are difficult to determine [14,15]. The MEMD is a novel phase-amplitude coupling measurement approach which couples a broadband, but it still suffers from mode mixing [16,17]. The masking EMD can effectively solve the mode mixing issue and suppress intermittent phenomenon in a transient process, but its mode separation ability is affected by the signal magnitude [18,19]. Besides, there are various other approaches for bearing element bearing fault diagnosis [20,21,22], but most of these methods mainly focus on noise reduction and ignore the inherent modulation characteristics of the vibration signal.

The modulation signal bispectrum (MSB) has emerged in fault detection because it can effectively utilize modulation characteristics along with high performance on noise suppression. In addition, it also has the ability to maintain phase information retention properties. Gu et al. [23] presented a new analysis approach to diagnose broken rotor bar faults by applying the MSB. Zhang et al. [24] applied the MSB to monitor the extent of gear wear deterioration. The results proved the feasibility of the MSB analysis approach in the fault detection of current signal analysis. Tian et al. [25] developed the MSB detector and successfully applied it to vibration signature analysis and produced more accurate detection results. Subsequently, Guo et al. [26] put forward a hybrid approach combined with wavelet packet energy (WPE) and MSB to detect bearing faults. However, these works assume that the original signal is stationary and may lead to some interfering frequency components when extracting fault features.

Ensemble empirical mode decomposition (EEMD) is an advanced nonlinear and non-stationary signal processing approach proposed by Wu and Huang [27], which has attracted wide attention in the fault detection of rolling element bearings [28,29,30,31]. Park et al. [28] used the EEMD method to classify gear tooth spall and crack defects. Shi et al. [29] developed a hybrid bearing fault detection method based on the adaptive stochastic resonance and analytical mode decomposition-EEMD. Fu et al. [30] presented a novel fault feature approach combining EEMD and the Elman_AdaBoot model. Amirat et al. [31] put forward a novel method for detecting motor bearings in combination with EEMD and statistical parameters. Although the EEMD has proven to be widely used for fault detection, how to choose the most representative intrinsic mode functions (IMFs) is still a tricky issue. Some analytical approaches for selecting most representative IMFs have recently been reported. For example, Chen et al. [32] developed a method for selecting sensitive IMFs using signal-to-noise ratio (SNR). Lei et al. [33] calculated the correlation coefficient between each IMF and the fault signal to choose sensitive IMFs. Xue et al. [34] introduced an effective IMF selection method based on kurtosis. Singh et al. [35] used Jensen Rényi divergence to adaptively select representative IMFs. Based on the scale of structural element (SE), Osman et al. [5] developed a novel approach for selecting effective IMFs. These methods are effective in selecting representative IMFs, but they do not take into account variations in impulse signal amplitude and instantaneous frequency, which may affect the accuracy of fault diagnosis. To solve this issue, Deng et al. [36] proposed using Teager energy kurtosis (TEK) to select sensitive IMFs. And it has been certified to be an effective approach to select representative IMFs [37]. Therefore, in view of the advantages of TEK, it is used to acquire representative IMFs in this paper. However, the above methods focus on analyzing individual IMF to extract fault features without considering the useful fault information that is usually omitted in discarding IMFs.

Considering all the above, a novel approach based on the weighted average ensemble empirical mode decomposition (WAEEMD) and the MSB for rolling element bearing fault diagnosis is proposed in this research work. Firstly, the complicated non-stationary signals are decomposed into a list of stationary intrinsic mode functions (IMFs) using EEMD. Subsequently, considering the effectiveness of different IMFs in revealing fault characteristics to avoid losing useful fault information, the weighted average method based on Teager energy kurtosis (TEK) is developed for signal reconstruction. Finally, the MSB is applied to the reconstructed signal using WAEEMD to decompose the modulation components for the fault characteristic frequencies identification and fault feature extraction.

The rest of the paper is organized as follows. Section 2 presents the brief principles of the MSB and its applications. Section 3 describes the mathematic model of the WAEEMD. Section 4 introduces the system framework of the proposed WAEEMD-MSB for bearing fault detection. Section 5 applies the proposed WAEEMD-MSB approach to analyze experimental signals for diagnosing a motor bearing outer race fault and a gearbox bearing inner race fault. Section 6 draws the conclusions based on the analysis results of the studies.

## 2. Modulation Signal Bispectrum

Modulation signal bispectrum (MSB) is an advanced signal demodulation method based on the improvement of the conventional bispectrum [23]. For a discrete-time signal x(t) with corresponding discrete Fourier transform (DFT) X(f), the MSB can be defined in the frequency domain as [24]:

(1) $$B_{MS}\left(f_{1},f_{2}\right)=E<X\left(f_{1}+f_{2}\right)X\left(f_{1}-f_{2}\right)X^{*}\left(f_{1}\right)X^{*}\left(f_{1}\right)>$$

where $B_{MS}\left(f_{1},f_{2}\right)$ and E<> represent the modulation signal bispectrum of signal x(t) and the expectation operator. The $X^{*}\left(f_{1}\right)$ means the complex conjugate of $X\left(f_{1}\right)$. The $f_{1}$ and $f_{2}$ denote the carrier frequency and modulating frequency, $\left(f_{1}+f_{2}\right)$ and $\left(f_{1}-f_{2}\right)$ mean the higher and lower sideband frequencies, respectively.

To more accurately quantize the sideband amplitude, MSB is improved by magnitude normalization to remove the effect of the carrier frequency of $f_{1}$. The MSB sideband estimator (MSB-SE) can be defined as [25]:

(2) $$B_{MS}^{SE}\left(f_{1},f_{2}\right)=\frac{B_{MS}\left(f_{1},f_{2}\right)}{\sqrt{\left|B_{MS}\left(f_{1},0\right)\right|}}$$

where $B_{MS}\left(f_{1},0\right)={\left|X\left(f_{1}\right)\right|}^{4}$ is the squared power spectrum estimation when $f_{2}=0$.

To get more robust results, the MSB detector is modified by the average of a few suboptimal MSB slices:

(3) $$B\left(f_{2}\right)=\frac{1}{N}\overset{N}{\underset{n=1}{\sum }}B_{MS}^{SE}\left(f_{1}^{n},f_{2}\right)\;,\;\left(f_{2}>0\right)$$

where *N* denotes the total number of selected suboptimal slices of *f*_1_, the number of which relies on the significance of the peaks themselves.

**Figure 1:**
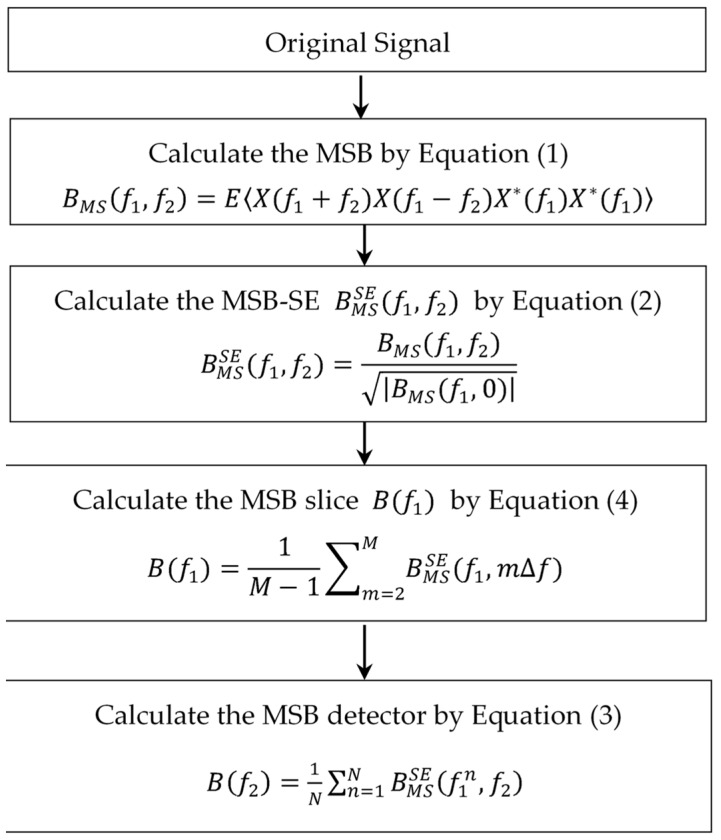
The flowchart of the modulation signal bispectrum (MSB) detector.

In order to obtain suboptimal slices of $f_{1}$, $B_{MS}^{SE}\left(f_{1}^{n},f_{2}\right)$ can be defined as the compound MSB slice $B\left(f_{1}\right)$, which is computed by averaging the significant MSB peaks in the incremental direction of the $f_{2}$ [38]:

(4) $$B\left(f_{1}\right)=\frac{1}{M-1}\overset{M}{\underset{m=2}{\sum }}B_{MS}^{SE}\left(f_{1,}m\Delta f\right)$$

where Δf represents the frequency resolution in the $f_{2}$ orientation, and M is the number of the significant MSB peaks. In summary, the flowchart of the MSB detector is shown in Figure 1.

## 3. Weighted Average Ensemble Empirical Mode Decomposition

Ensemble empirical mode decomposition (EEMD) is a self-adaptive method for decomposing non-stationary and non-linear signal. It was first proposed by Huang et al. [27], which can adaptively decompose complex vibration signal into a list of different IMFs.

(5) $$y\left(t\right)=\overset{N}{\underset{i=1}{\sum }}IMF^{i}\left(t\right)+r_{N}\left(t\right)$$

where y(t) is the analyzed signal, $IMF^{i}\;\left(i\;=1,\;2,\ldots ,N\right)$ is the *i*-th IMF, and N is the number of IMFs decomposed by EEMD.

By using EEMD, the vibration signal is separated into a set of IMFs. A number of IMFs are expected to involve the most fault information related to the health of rotating machinery, and IMFs may not be missed during the monitoring process. In order to avoid losing useful fault information, the effectiveness of different IMFs in revealing fault characteristics is considered, the weighted average method based on Teager energy kurtosis (TEK), namely WAEEMD, is proposed. The WAEEMD can be expressed as follows:

(1) Calculate the TEK value of the $IMF^{i}$ based on Equation (6) as [39]:

(6) $$TEK=\frac{\left(M-1\right)\sum_{t=1}^{M}{\left(\varphi_{IMF^{i}}\left(t\right)-{\bar{\varphi }}_{IMF^{i}}\right)}^{4}}{{\left(\sum_{t=1}^{M}{\left(\varphi_{IMF^{i}}\left(t\right)-{\bar{\varphi }}_{IMF^{i}}\right)}^{2}\right)}^{2}}$$

where $\varphi_{IMF^{i}}\left(t\right)\;\left(t=1,2,\cdots ,\;M\right)$ indicates the Teager energy of $IMF^{i}$ at the sample time t, and ${\bar{\varphi }}_{IMF^{i}}$ stands the mean value of $\varphi_{IMF^{i}}\left(t\right)$.

(2) Calculate the weighted average coefficient w(i) that can be expressed as:

(7) $$w\left(i\right)=e\left(i\right)/\overset{N}{\underset{i=1}{\sum }}e\left(i\right)$$

where $e\left(i\right)=TEK^{i}$ represents the TEK value of each $IMF^{i}$.

(3) Calculate the final output result by the WAEEMD:

(8) $$IMF_{w}^{i}=\overset{N}{\underset{i=1}{\sum }}w\left(i\right)IMF^{i}$$

## 4. The Procedure of the WAEEMD-MSB

**Figure 2:**
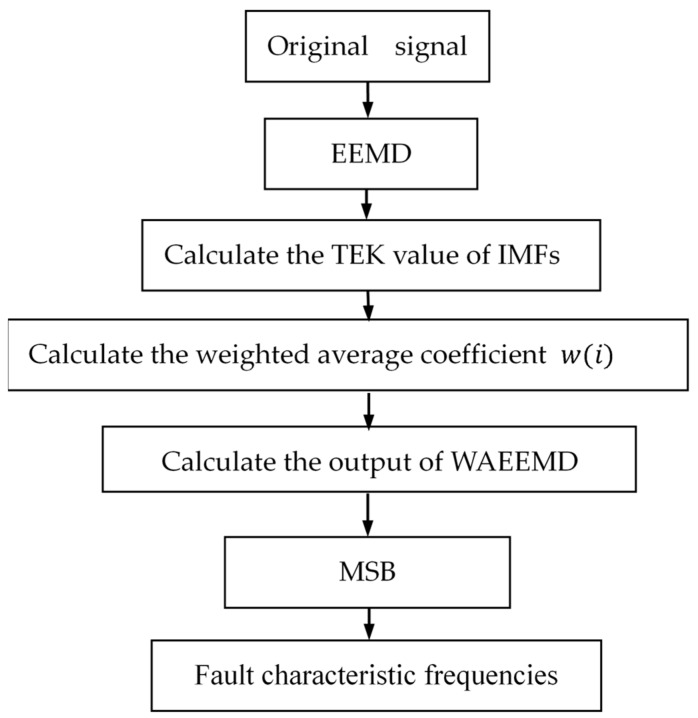
The flowchart of the weighted average ensemble empirical mode decomposition (WAEEMD)-MSB.

Motivated by the advantages of WAEEMD and MSB, this paper developed the proposed WAEEMD-MSB for the fault detection of rolling element bearings. The detailed procedures of the WAEEMD-MSB are illustrated in Figure 2 and summarized as follows:

Step 1: Decompose the original signal into a few series of IMFs using EEMD;
Step 2: Calculate the TEK value by Equation (6) in different decomposition levels of EEMD;
Step 3: Acquire the reconstructed signal by the WAEEMD involving the most representative IMFs;
Step 4: Perform MSB to the reconstructed signal for fault characteristic frequencies extraction.

## 5. Experimental Validation

To verify the effectiveness performance of the proposed WAEEMD-MSB for bearing fault diagnosis, experimental signals collected from a 3-phase induction motor with bearing outer race fault and a 2-stage helical gearbox with bearing inner race fault were carried out for the experimental validation. Additionally, to illustrate the advantages of the WAEEMD-MSB, the analysis results of the WAEEMD-MSB were compared with the conventional MSB and EEMD-MSB analysis methods.

### 5.1. Description of the Experiments

**Figure 3:**
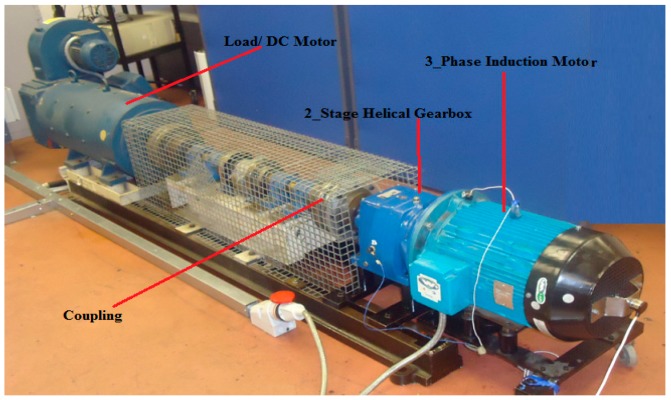
Rolling element bearing test rig.

**Figure 4:**
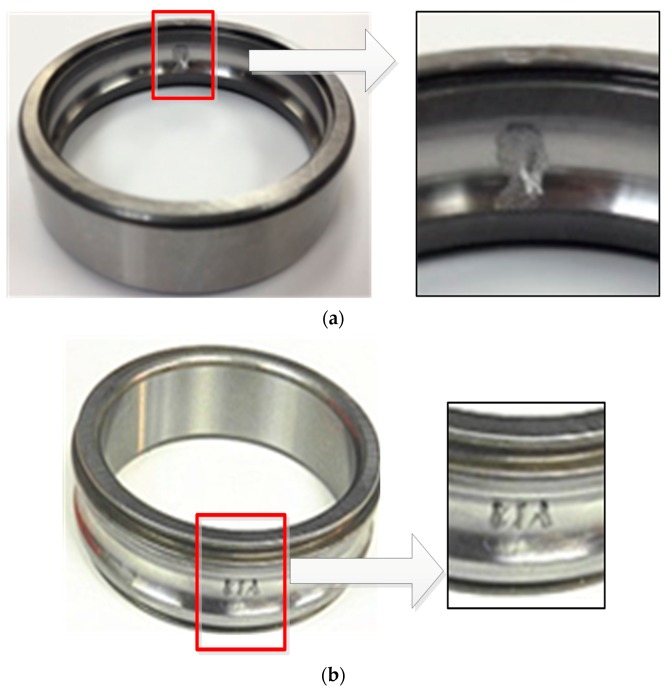
The faulty bearing: (**a**) outer race fault; (**b**) inner race fault.

The rolling element bearing test rig and its photograph are depicted in Figure 3. The test rig was comprised of a DC load motor, 3-phase induction motor, 2-stage helical gearbox, flexible couplings, and some accelerometers. In the experiment, one accelerometer was positioned on the vertical direction of the 3-phase induction motor drive end bearing housing, and the other accelerometer was glued on the 2-stage helical gearbox housing as shown in Figure 3. The faulty bearings were setup to two type of faults, one was an outer race fault in the bearing of the 3-phase induction motor, and the other was an inner race fault in the bearing of the 2-stage helical gearbox. The faults in the outer and inner race were shown in Figure 4, respectively. The structure parameters of faulty bearings are listed in Table 1.

### 5.2. Diagnosis Results for the Bearing Inner Race Fault

**Figure 5:**
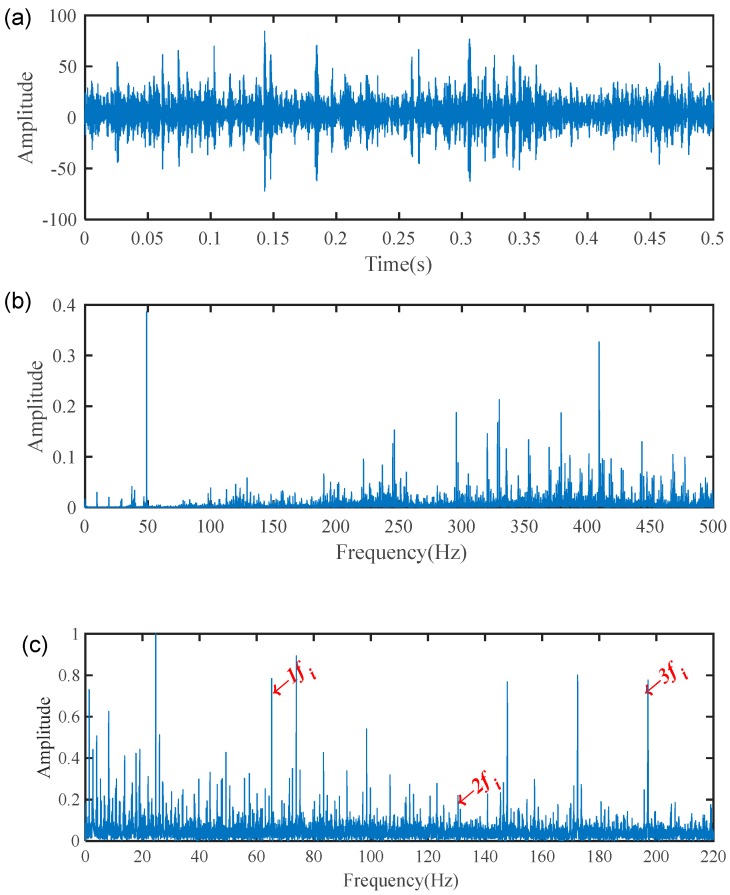
Vibration signal of the 2-stage helical gearbox bearing with inner race fault: (**a**) waveform; (**b**) spectrum; (**c**) envelope spectrum.

The vibration signals from the tested 2-stage helical gearbox bearings were collected at a sampling frequency of 96 kHz with a shaft speed around 1500 rpm. The theoretical shaft rotational frequency $f_{r}$ and fault characteristic frequency $f_{i}$ are about 25 Hz and 65.17 Hz, respectively. Figure 5 shown the waveform, spectrum, and envelope spectrum of the measured vibration signal. It can be seen that the measured signal is very complex and the useful information for fault identification is hidden by the heavy noise. It is difficult to recognize the fault characteristic frequency $f_{i}$ from Figure 5b. Then, the envelope analysis was applied to analyze the measured signal shown in Figure 5a. The fault characteristic frequencies were extracted directly, but there was still a large of background noise and interference components as shown in Figure 5c.

**Figure 6:**
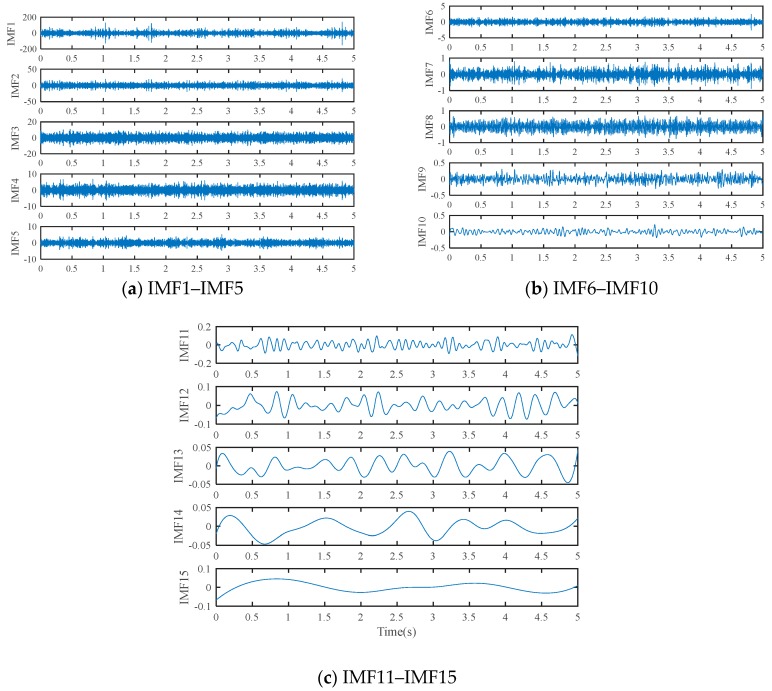
Results of fault signal analyzed by ensemble empirical mode decomposition (EEMD).

**Figure 7:**
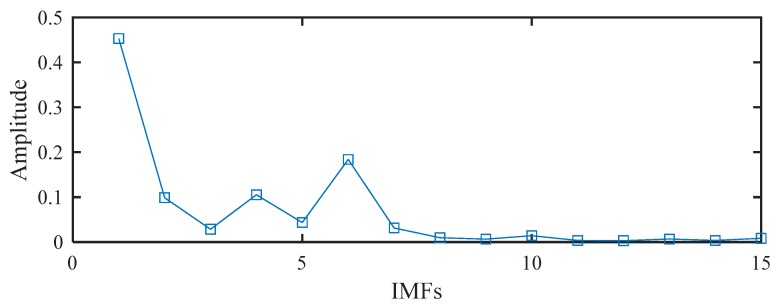
The weighted average coefficients using Teager energy kurtosis (TEK).

**Figure 8:**
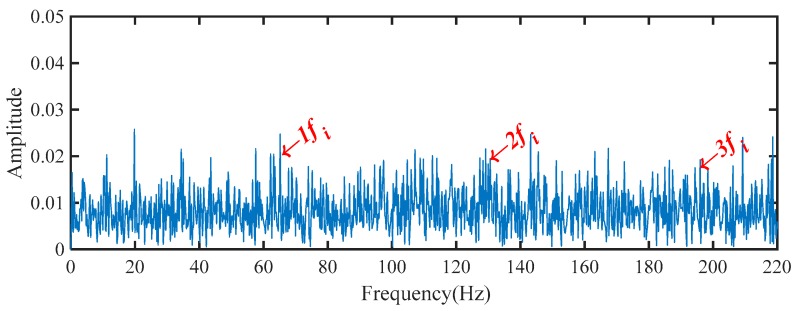
The spectrum of the WAEEMD filtered signal.

Based on the proposed method, the measured vibration signals in Figure 5a were firstly decomposed into 15 IMFs by the EEMD, as shown in Figure 6. Considering the effectiveness of each IMF in revealing fault characteristics, the weighted average coefficients based on TEK were calculated and displayed in Figure 7. The spectrum of processed signal by the WAEEMD was shown in Figure 8. Obviously, it cannot effectively extract the fault characteristic frequency $f_{i}$ and its harmonics due to strong heavy noise and many interference components.

**Figure 9:**
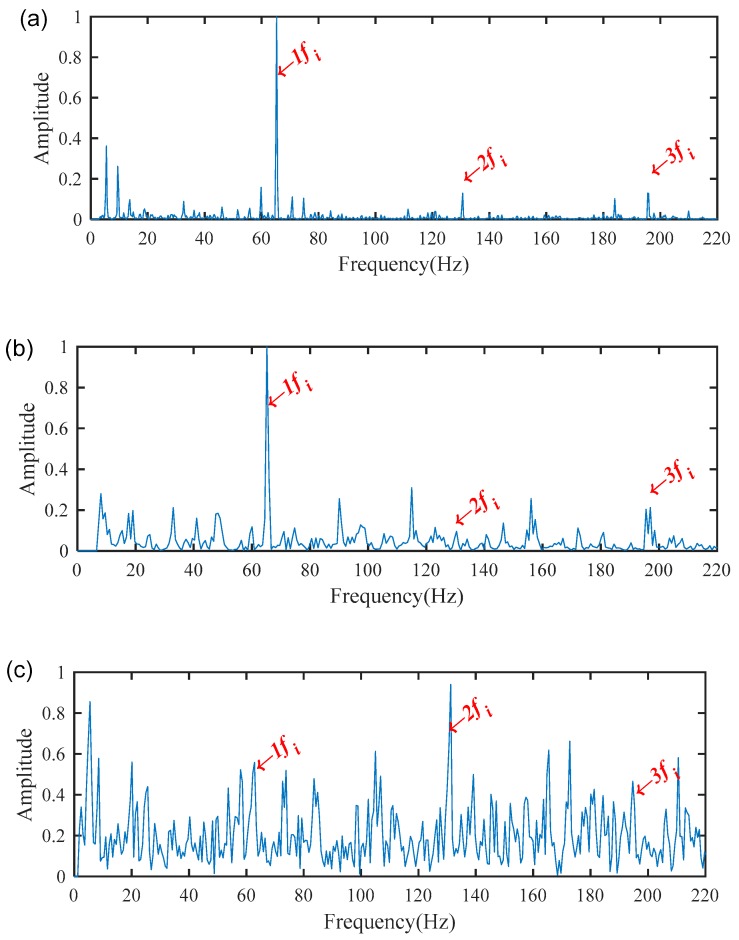
Diagnosis results for the bearing inner race fault using: (**a**) WAEEMD-MSB; (**b**) conventional MSB; (**c**) EEMD-MSB.

The MSB was then used to process the WAEEMD filtered signal and decompose the modulated components and suppress heavy noise, thereby extracting fault characteristic frequencies, as illustrated in Figure 9a. It can be clearly seen that the fault characteristic $f_{i}$ and its harmonics were recognized based on the analysis results by the proposed method. Additionally, the conventional MSB without the pre-processed using WAEEMD and individual most sensitive IMF (the 1st IMF with highest TEK value as shown in Figure 7) of EEMD combined with MSB (EEMD-MSB), were also applied to process the raw vibration signal in Figure 5a for comparison. The spectrum of the conventional MSB was mixed with heavy noise, and some interference harmonics still exists, as shown in Figure 9b. For EEMD-MSB, IMF1 was selected as the most sensitive IMF from Figure 6, because it has the largest Teager energy kurtosis (TEK) index value compared with other IMFs as shown in Figure 7, which indicating that it contains the most fault characteristics information. The MSB spectrum of the IMF1 is shown in Figure 9c. By comparison with Figure 9a, it is impossible to obtain accurate fault features based on the diagnosis result by EEMD-MSB as illustrated in Figure 9c. This shows that the WAEEMD-MSB can achieve more accurate results in the diagnosis of the bearing faults than the conventional MSB and EEMD-MSB methods. To further explain the effectiveness of WAEEMD-MSB, an indicator named the characteristic frequency intensity coefficient (CFIC) was calculated to evaluate the performance of conventional MSB, EEMD-MSB, and WAEEMD-MSB, which is defined as follows [40]:

(9) $$CFIC=\frac{\sum_{k=1}^{m}Y\left(kf\right)}{\sum_{j=1}^{n}Y\left(f_{j}\right)}$$

where $Y\left(f_{j}\right)\;\left(j=1,2,\ldots ,n\right)$ denotes whole frequencies in the selected frequency band (0–220 Hz). Y(kf)(k=1,2,…,m) represents the amplitude at the k th harmonic of fault characteristic frequencies. The larger the CFIC value, the better the filtering effect by using denoising method.

Table 2 indicates the CFIC of conventional MSB, EEMD-MSB, and WAEEMD-MSB on the tested 2-stage helical gearbox bearings. It demonstrated that the WAEEMD-MSB can extract fault features well and greatly reduce background noise. In conclusion, these results indicated that WAEEMD-MSB is more effective than conventional MSB and EEMD-MSB.

### 5.3. Diagnosis Results for the Bearing Outer Race Faults

**Figure 10:**
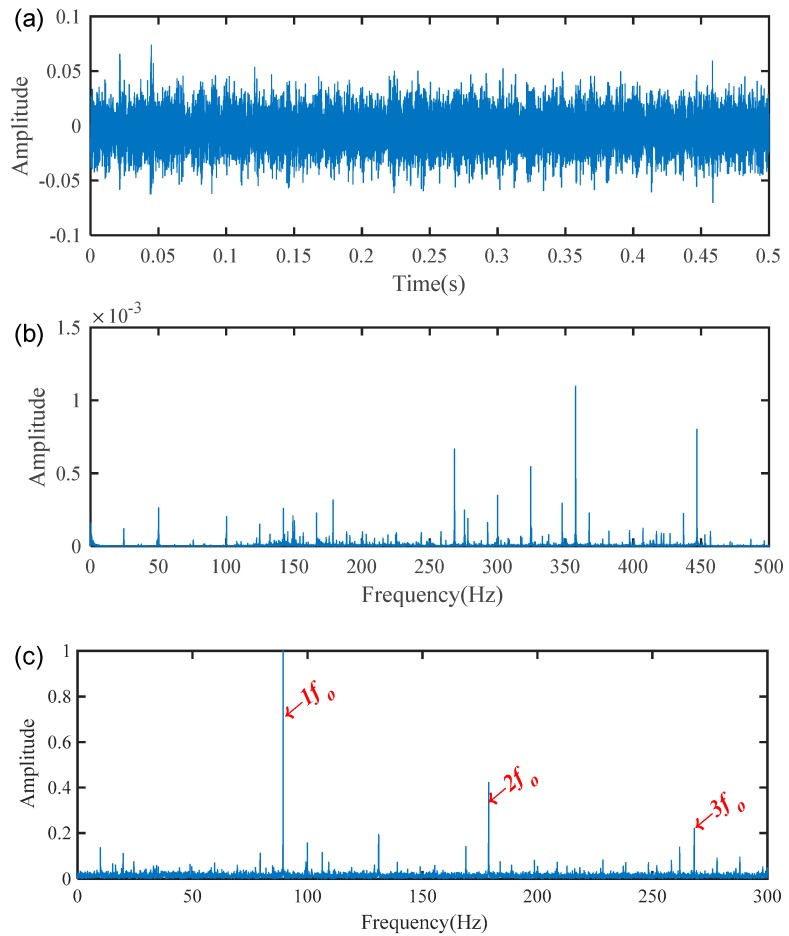
Vibration signal of the bearing with outer race fault: (**a**) waveform; (**b**) spectrum; (**c**) envelope spectrum.

The sampling frequency of the measured vibration signal was 96 kHz and the test rig was operated at around 1500 rpm, respectively. Therefore, the theoretical shaft rotational frequency $f_{r}$ and fault characteristic frequency for the bearing outer race faults $f_{o}$ are 25 Hz and 89.38 Hz, respectively. The waveform, corresponding spectrum, and envelope spectrum of the measured vibration signal are illustrated in Figure 10. It can be seen that the measured signal was very complex, and the fault characteristic frequencies were hidden by the strong background noise. The fault characteristic frequency $f_{o}$ and its harmonics cannot be directly extracted from the spectrum in Figure 10b. According to the envelope spectrum in Figure 10c, the fault characteristic frequency and its harmonics can be identified, but there are also some interference components, especially around the higher order harmonics ($3f_{o}$).

**Figure 11:**
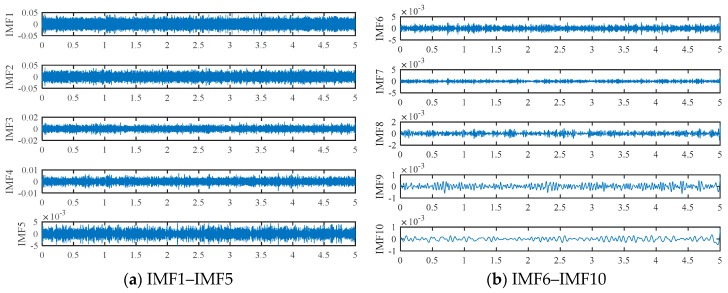

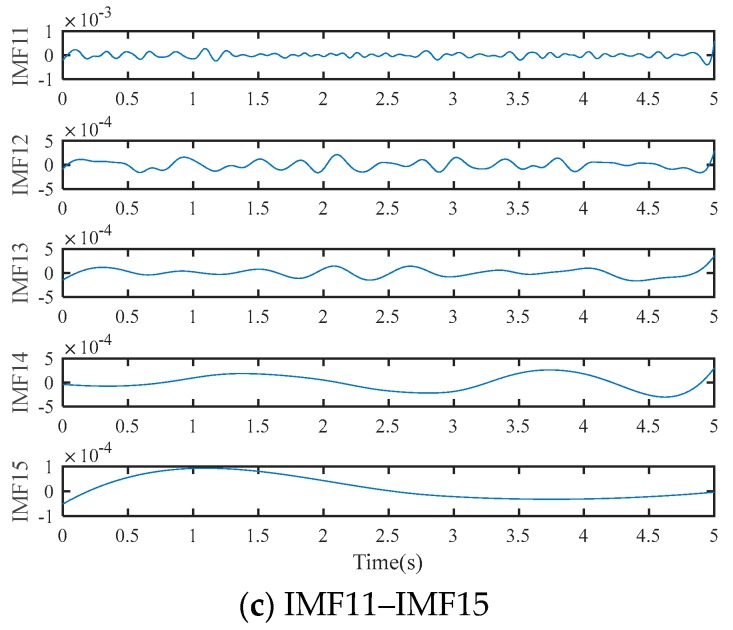
Results of fault signal analyzed by EEMD.

**Figure 12:**
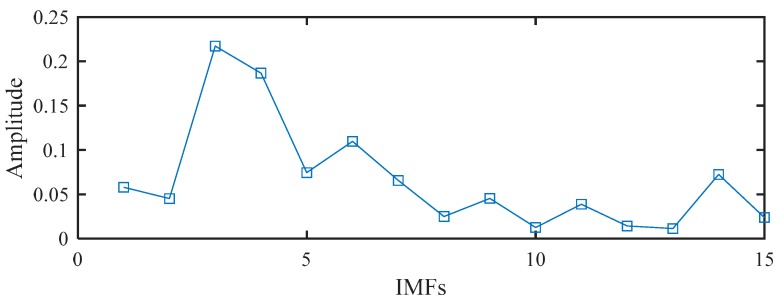
The weighted average coefficients using TEK.

**Figure 13:**
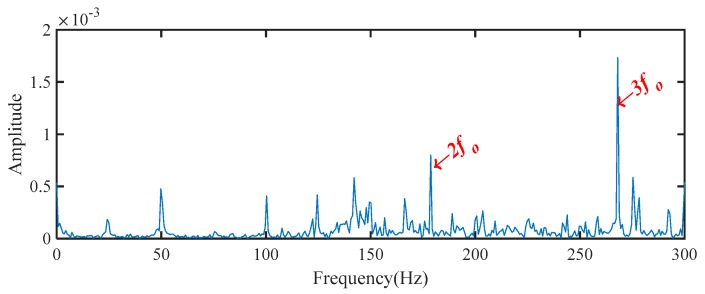
The spectrum of the WAEEMD filtered signal.

The WAEEMD-MSB was then employed to analyze the vibration signal shown in Figure 10a. The signal was initially decomposed into 16 IMFs by EEMD as shown in Figure 11 (The last residual is not displayed). The weighted average coefficients were then calculated and are shown in Figure 12. Based on the spectrum of the processed signal by WAEEMD shown in Figure 13, it can be seen the higher-frequency harmonics was mixed with noise and many interference components, and the fault characteristic frequency $f_{o}$ cannot be identified effectively.

**Figure 14:**
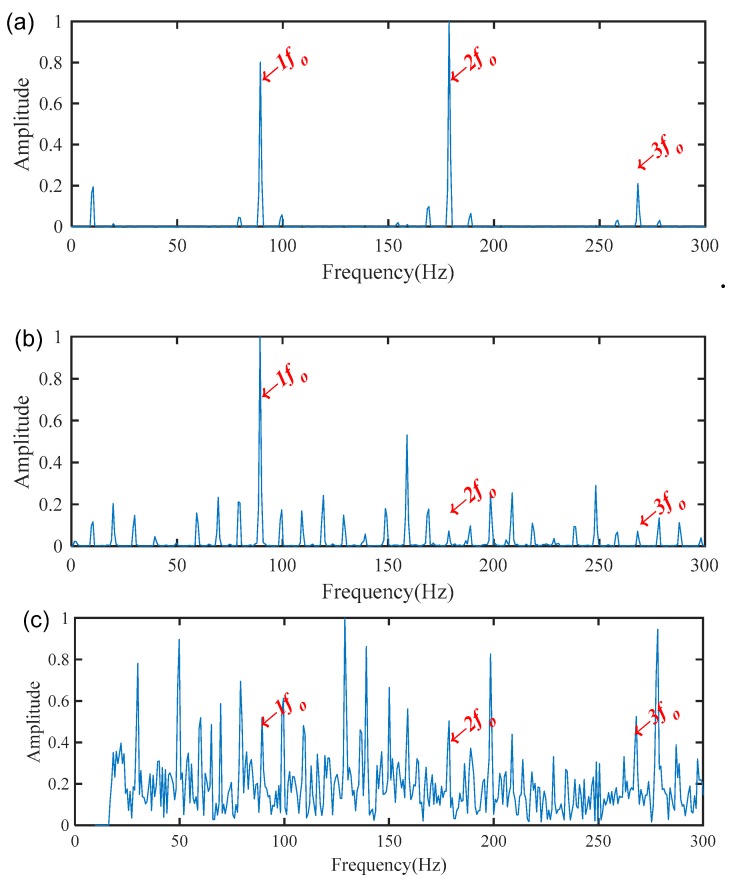
Diagnosis results for the bearing outer race fault using: (**a**) WAEEMD-MSB; (**b**) conventional MSB; (**c**) EEMD-MSB.

The MSB was then used to analyze the processed signal by WAEEMD and the diagnosis result is illustrated in Figure 14a. For comparative analysis, the conventional MSB, without the pre-processing using WAEEMD and the individual most sensitive IMF (the 3rd IMF with highest TEK values as shown in Figure 12) of EEMD combined with MSB (EEMD-MSB), were also applied to process the measured signal shown in Figure 10a and the analysis results are shown in Figure 14b,c respectively. Obviously, the spectrum based on the conventional MSB analysis can reflect the fault characteristic frequencies, but the interference frequency components also appear in Figure 14b, while the spectrum of the EEMD-MSB analysis result was mixed with a lot of heavy noise, that can be seen in Figure 14c. By contrast, Figure 14a it clearly shows that the WAEEMD-MSB can recognize and extract the fault characteristic frequency $f_{o}$ and its harmonics with higher accurately and effectively. The CFIC of fault characteristic frequencies in the frequency band of 0–300 Hz extracted by conventional MSB, EEMD-MSB, and WAEEMD-MSB methods were calculated and illustrated in Table 3. It is clear that EEMD-MSB is more effective than conventional MSB, EEMD-MSB for fault feature extraction from the vibration signal.

## 6. Conclusions

In this paper, a novel fault detection approach based on WAEEMD and MSB analysis was proposed. Based on the validation using two case studies for the bearing outer race and inner race fault diagnosis, the conclusions can be drawn as follows:

(1) The weighted average coefficients based on Teager energy kurtosis (TEK) has the ability to highlight the representative IMFs and reducing the disturbance of the IMFs that has less correlation with faults;
(2) WAEEMD can effectively solve the weakness of MSB when dealing with non-stationary signals, and further enhance the performance and accuracy of fault feature extraction;
(3) MSB has advantages of decomposing the modulated components and suppressing the noise of the processed signal by WAEEMD for fault feature extraction;
(4) The experimental signals are measured from the defective bearings to assess the feasibility and effectiveness of the proposed WAEEMD-MSB approach. The analysis results demonstrate that the proposed WAEEMD-MSB can produce more accurate fault features when compared to conventional MSB and EEMD-MSB in the fault diagnosis of rolling element bearing.

## Figures and Tables

**Table 1:** The structural parameters of the faulty bearing.

Bearing Model	Ball Diameter *d* (mm)	Pitch Diameter $D_{m}$ (mm)	Ball Number *z*	Contact Angle β
6008	7.9	54	12	$0^{{}^{\circ}}$
6206ZZ	9.53	46.4	9	$0^{{}^{\circ}}$

**Table 2:** The characteristic frequency intensity coefficient (CFIC) of conventional MSB, EEMD-MSB, and WAEEMD-MSB.

Methods	CFIC
MSB	1.24%
EEMD-MSB	0.15%
WAEEMD-MSB	3.66%

**Table 3:** The CFIC of conventional MSB, EEMD-MSB, and WAEEMD-MSB.

Methods	CFIC
MSB	9.53%
EEMD-MSB	3.65%
WAEEMD-MSB	26.62%
